# A guide on transnasal endoscopy: setting up a pediatric unsedated endoscopy program

**DOI:** 10.3389/fped.2023.1267148

**Published:** 2024-01-16

**Authors:** Joel A. Friedlander, Kristina Leinwand, Vrinda Bhardwaj, Nathalie Nguyen

**Affiliations:** ^1^ EvoEndo, Inc., Centennial, CO, United States; ^2^ Pediatric Gastroenterology, Northwest Permanente/Kaiser Permanente Physicians & Surgeons, Portland, OR, United States; ^3^ Division of Pediatric Gastroenterology, Oregon Health & Sciences University, Portland, OR, United States; ^4^ Division of Gastroenterology, Hepatology and Nutrition, Children’s Hospital Los Angeles, Los Angeles, CA, United States; ^5^ University of Southern California, Los Angeles, CA, United States; ^6^ Gastrointestinal Eosinophilic Diseases Program, Section of Pediatric Gastroenterology, Hepatology and Nutrition, University of Colorado School of Medicine; Digestive Health Institute, Children’s Hospital Colorado, Aurora, CO, United States

**Keywords:** transnasal endoscopy, sedation free, pediatrics, pediatric gastroenterology and hepatology, endoscopy unit

## Abstract

**Background:**

Unsedated transnasal endoscopy is becoming an increasingly popular option for the evaluation of upper gastrointestinal tract disorders in adults and children worldwide. This innovative technology has transformative potential as it provides for a more efficient, safe, and cost-effective method for endoscopy and reduces the risks associated with anesthesia, which is particularly relevant in pediatrics as endoscopy is commonly done under general anesthesia or conscious sedation. The aim is to address knowledge gaps amongst pediatric gastroenterologists who may be considering the development of a TNE program, detailing how to implement sedation-free TNE into practice for pediatric patients and current and forthcoming technologies.

**Methods:**

We conducted a comprehensive review of current literature and collection of data from experts and clinicians in the field on how sedation-free programs were started and being conducted. We aimed to collate the data to provide a guide to address knowledge gaps with a focus on setting up and starting a sedation-free endoscopy program.

**Results:**

Here in, we provide a detailed guide for implementing a sedation-free endoscopy program in pediatrics including design and layout of a TNE unit, special staffing needs, equipment, current and forthcoming technologies, financial considerations and training considerations. We highlight special considerations that are relevant in pediatrics incorporating distraction or dissociation techniques such as Virtual Reality Systems, developmentally appropriate preparation for children, and topical analgesia.

**Conclusion:**

Sedation-free endoscopy is a rapidly growing option for pediatric patients. Development of an unsedated pediatric endoscopy program will improve patient care, decrease the need for anesthesia, provide a lower cost and safe alternative to traditional sedated endoscopy, and is a viable component to a pediatric gastroenterology practice.

## Introduction

Unsedated transnasal endoscopy (TNE) with virtual reality (VR) distraction dissociative technology has been described increasingly at a few pediatric centers over the past several years (1–5). In pediatric gastroenterology, sedation-free transnasal endoscopy developed from a collaboration with pulmonology and otolaryngology. It encompasses a series of procedures that includes transnasal esophagoscopy (TN-Eso), transnasal gastroesophagoscopy (TN-EG), or transnasal esophagogastroduodenoscopy (TN-EGD). This innovative technology has transformative potential in the monitoring of pediatric gastrointestinal disease. It provides for a more efficient, safer, and cost-effective method to diagnose and evaluate pediatric disorders of the upper gastrointestinal (GI) tract (Nguyen et al, Sabe et al). In pediatrics, esophagogastroduodenoscopy (EGD) is most commonly done under general anesthesia or conscious sedation. Previous studies suggest the overall complication rate in pediatrics of EGD under general anesthesia or conscious sedation of 2.3%, while unsedated TNE eliminates the potential risks of anesthesia (Thakkar et al. PMID 17258979). When comparing EGD to TNE, previous studies highlight a 50% charge reduction with TNE, owing largely to charges associated with anesthesia and decreased total visit time for endoscopy (163 min for EGD vs. 36 min for TNE) leading to decrease time away from work and school for patients and families (Nguyen et al. PMID: 30708107, Sabe et al). For these reasons, there has been growing interest in TNE in pediatrics.

TNE has been utilized to assess many upper GI tract diseases and a variety of channeled or unchanneled devices. Many devices have since been taken off the market and some remain. In pediatrics, the indications TNE primarily involved biopsy and have been described to include eosinophilic esophagitis, dysphagia, candida esophagitis, abdominal pain, gastroesophageal reflux disease, and celiac disease (PMID: 30708107). Additionally, adult gastroenterologists have utilized TNE for a broader list of indications that may involve only visualization or biopsy including globus pharyngeus, Barrett's esophagus, gastroesophageal reflux disease, esophageal varices, and gastric carcinoma (PMID 12556784).

Herein, we describe the ideal method and process for pediatric gastroenterologists to establish a pediatric TNE program. Though adult literature may provide insight, the adult methods vary from the recently introduced pediatric concept (6–8). For example, the pediatric method is usually performed in an ambulatory clinic room in a sitting position, commonly uses VR distraction and dissociation with assistance from child-life support staff, as compared to the adult method of unsedated endoscopy which may utilize an ambulatory surgery center while the patient is in lateral recumbent position. The aim is to address knowledge gaps amongst pediatric gastroenterologists who may be considering the development of a TNE program, detailing how to implement sedation-free TNE into practice for pediatric patients and current and forthcoming technologies.

### Methods

We conducted a comprehensive review of current literature and collection of data from experts and clinicians in the field on how sedation-free programs were started and being conducted. We aimed to collate the data to provide a guide to address knowledge gaps with a focus on setting up and starting a sedation-free endoscopy program. The authors represent multiple institutions including academic children's hospitals and community-based practices.

## Designing the TNE unit

### Ambulatory TNE unit design

The TNE unit varies from an ambulatory surgery center, procedure center, or operative environment as the goal is to avoid the anxiety provoking concept of perioperative care. The pediatric TNE unit is typically located in the outpatient clinic area in a designated procedure room, which can be a custom unit or an existing space. Workflow should accommodate an efficient check-in area as in a clinical setting, a pre-procedure room near the procedure room, and storage room for supplies and cleaning equipment. A code cart should be available in close proximity to provide for emergency care if needed. Attention to anxiety-decreasing concepts is recommended to help patients and families feel comfortable. When starting a program, sharing or coordinating a workspace with other specialties such as pulmonology or otolaryngology could be beneficial to decrease the budget implications.

### Preparation or pre-procedure room (clinic room)

The procedure preparation room is where the patient is roomed to after check-in. It is often a standard pediatric clinic room that is used for routine clinic visits. This room however may require some additional materials and supplies. These include an area for charging virtual reality (VR) equipment and its associated accessories or a UV sterilizer for VR if a reusable system is used. A computer system and/or tablet computer is recommended to help facilitate selection of distraction or media materials for preparation. Locking storage cabinets are also recommended for VR equipment, as these can be expensive pieces of equipment.

### Procedure room

**Figure 1:**
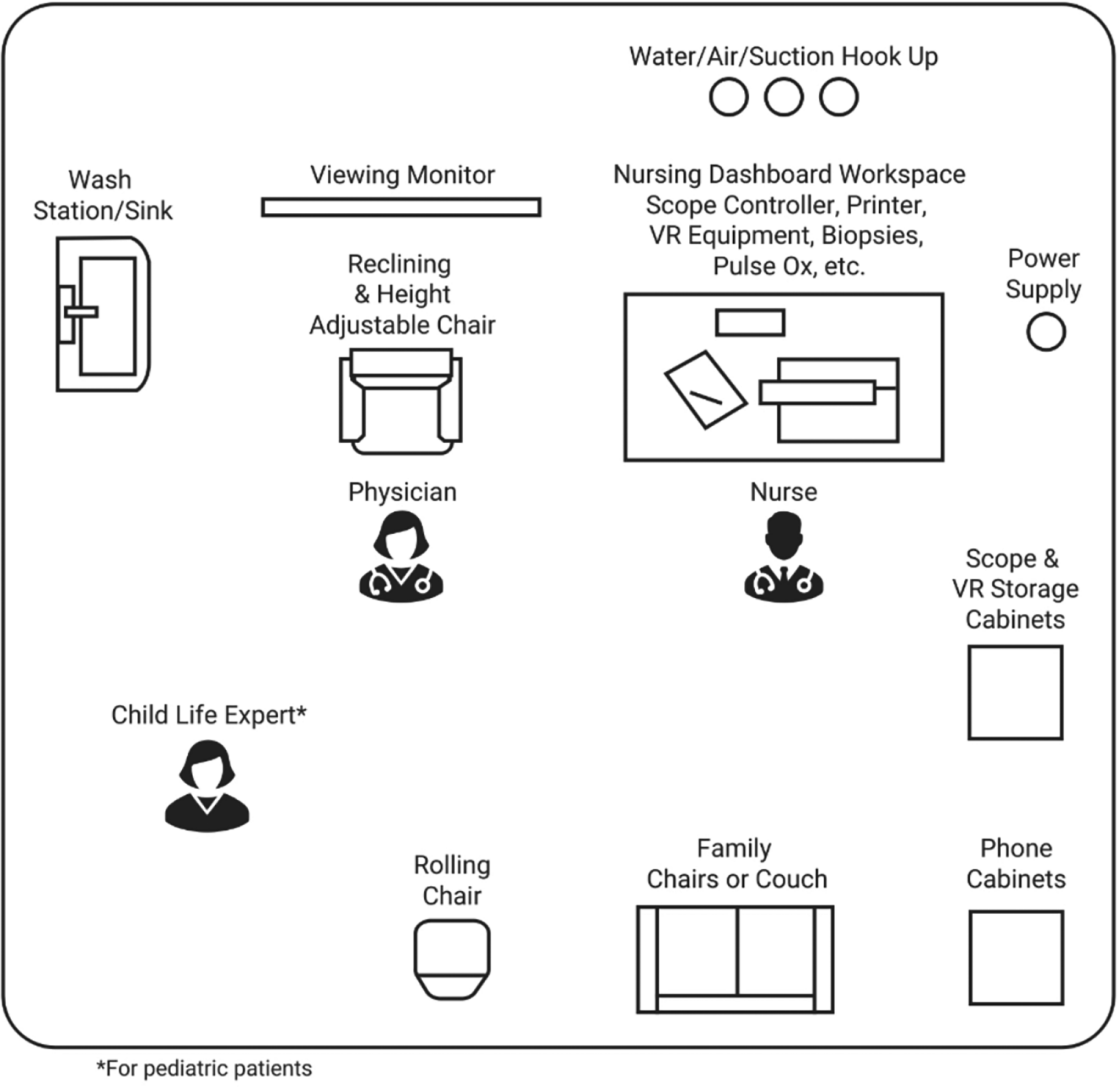
Example of procedure room. *Diagram supplied by EvoEndo, Inc.

The procedure room should be cool and well lit. It should have adequate ventilation and wall sources for compressed air/carbon dioxide, oxygen, and suction for use during the procedure or in case of an emergency. The room should be a patient/family friendly room to decrease anxiety and increase comfort. It should have adequate cabinet space for supplies. The room should have an adjustable chair designated for procedures for the patient (has head, foot and arm rest and has the ability to recline and adjust the height of the chair). The procedure is commonly done with parents present, therefore, a comfortable chair for family members is recommended. The room should be set up with the patient chair across from the chair the parent is sitting in to allow for parental viewing of the screen and to allow them to be close to their child for calming assistance. Young patients and those with anxiety may benefit from holding their parent's hand for support, therefore the ability for close proximity of chair the parent is sitting in to the patient is important. The room should also allow for the presence of a nursing work station, an endoscopy station, and a table for supplies needed during the procedure. Some centers perform transnasal endoscopy in a procedure unit room or operating room while others perform in an ambulatory outpatient clinic area. The setup of an ideal TNE procedure room is demonstrated in Figure 1.

### Endoscope and VR equipment processing area

Depending on the equipment, there are different needs for post equipment processing. If TNE and VR equipment is single use then an associated disposal area for sharps and medical device disposal or recycling is needed. If the endoscope is reusable and requires reprocessing, an additional room for the cleaning is required with all associated plumbing, electrical, staff work area, and storage. Endoscope reprocessing will vary based on if biopsies are required at the time of endoscopy. This process is the same processing as for endoscopy units. Some centers share reprocessing with the endoscopy unit, while others courier the endoscope for processing to a central sterile processing location after use. Preventing the spread of infection and endoscope reprocessing has becoming increasingly important amidst the COVID pandemic. Reusable VR goggles will also require cleaning between uses. Equipment for cleaning VR goggles could be located in the procedure room, pre/post procedure room, or a storage room.

The recommended capital supply list to start a TNE program is listed in Table 1A.

**Table 1:** Unsedated endoscopy program Recommended capital supply list & procedural supply list.

A. Capital Supply List
Endoscopes (2–4 reusable endoscopes recommended-if applicable)
Endoscopy cart (controller and medical grade endoscopy/surgical monitor)
Endoscopy cleaning equipment or disposal/recycling container
Moderately sized child-friendly room with adequate ventilation and dimmable lights
Reclining/height adjustable chair with armrests
Biohazard/sharp disposal
Air/CO2 supply
Suction supply
Bag valve mask
Code cart
Equipment for vital Signs including pulse oximeter
Table/Cart for supplies during endoscopy
Biopsy specimen light
Biopsy specimen tray
Endoscope storage if stored on site
Computer with reporting software (with image & video capture) and printer
Chair for family member
Virtual reality system (with Wi-Fi/commercial media subscription)
Virtual reality cleaning system^a^
Freezer for popsicles/post-procedure drinks^a^
B. Consumable Procedural Supply List
Single-Use Endoscopes (one-month supply recommended-if applicable)
Biopsy containers
Biopsy forceps
Towels
Air extension tubing
Suction extension tubing
Endoscope valves
Disposable table cover
Gloves and other PPE including procedure gown and eye shield
4% lidocaine spray and atomizer bottle
Oxymetazoline
Oxivir/cleaning wipes
10 ml slip tip syringe
Silicone spray or lubricant
Emesis bag
250–500 ml sterile water
Cleaning Supplies for endoscope and solidifier for suction canister
Water/specimen cup
Specimen labels
Popsicle or clear liquids

^a^ Optional Considerations.

## Unit management

### Staffing and unit coverage

The staffing of a pediatric unsedated endoscopy unit requires a smaller team than an ambulatory procedure unit as there are no preoperative and post-operative care areas. Having multiple staff in each role for backup and procedural access is recommended. The staffing for the units includes: 1) Gastroenterologist/Endoscopist performing TNE 2) Nursing 3) Check-in Staff 4) Child Life Specialist. Some centers may also include 5) Medical Assistant to help efficiency of equipment and room turnover.

### Staffing requirements/credentialing

A sedation-free endoscopy is subject to the same credentialing requirements as any other procedure in the hospital and approved by the appropriate committee. The requirements may need to be developed by the TNE team. Because this is a novel procedure and no one may be currently performing it at your center, credentialing may be a barrier. The authors were credentialed by physicians who performed unsedated laryngoscopy in order to learn the nasal evaluation and as gastroenterologists were already credentialed in esophagoscopy. Hospitals have specific requirements for credentialing and the authors defer to the institution's guidelines.

### Special staffing

Child Life specialists can provide developmentally appropriate support to aid with coping skills during healthcare encounters. They are trained in the developmental impact of illness and injury and help a child understand unsedated endoscopy. Child Life specialists can develop a series of handouts, tablet-based presentations and videos that are age appropriate which be very helpful in providing anticipatory guidance and preparing the child immediately before the procedure. These handouts and videos are specific for children to understand what to expect before, during and after TNE. Child Life specialists are especially helpful for first-time procedures or for patients with procedural anxiety, but may not be necessary for repeat TNE procedures, and locations with limited Child Life resources may be able to prioritize assistance accordingly.

## Equipment

### Endoscope systems

A major difference between pediatric and adult transnasal endoscopy is the outer diameter of the endoscope. Pediatric studies report the use of ultra-thin endoscopes that are 2.8–4.2 mm in outer diameter with a 1.2–2.0 mm biopsy channel while adult ultra-thin endoscopes (commonly referred as pediatric neonatal gastroscopes) are 4.9–5.8 mm in outer diameter with a 2.0–2.2 mm biopsy channel (Table 2). Depending on the endoscope, they have 2 or 4 way deflection, have a full array of endoscope functions and are commonly used internationally (9).

**Table 2:** List of available endoscopes in the United States for pediatric unsedated endoscopy.

Brand	Model	Max Outer Diameter (mm)	Working Length (cm)	Channel Size (mm)	Field of View (Degrees)	Steering	Buttons
AMBU	aScope 4 BronchoSlim	4.2	60	1.2	85	2-way	Air
AMBU	aScope 4 BronchoRegular	5.4	60	2.0	85	2-way	Air
AMBU	aScope 5 BronchoRegular	2.7	60	1.2	120	2-way (+rotary)	Air
AMBU	aScope 5 BronchoRegular	4.2	60	2.2	120	2-way (+rotary)	Air
AMBU	aScope 5 BronchoRegular	5.0	60	2.2	120	2-way (+rotary)	Air
Boston Scientific	Exalt Model B Slim	4.3	60	1.0	90	2-way	Air
Boston Scientific	Exalt Model B Regular	5.5	60	2.0	90	2-way	Air
Boston Scientific	Exalt Model B Large	6.3	60	2.6	90	2-way	Air
EvoEndo	Model LE	3.5	110	2.0	120	4-way	A/W/S
Fujinon	EG-530n	5.9	110	2.0	120	4-way	A/W/S
Fujinon	EG-530NP	5.1	110	2.0	120	2-way	A/W/S
Fujinon	EB-580S	5.3	60	2.2	120	2-way	Air
Fujinon	EB-530P	3.8	60	1.2	120	2-way	Air
Fujinon	EB-580T	5.9	60	2.8	120	2-way	Air
Fujinon	EB-530H	5.4	60	2.0	120	2-way	Air
Olympus	GIF-XP190	5.8	110	2.2	140	4-way	A/W/S
Olympus	N190	4.9	110	2.0	120	2-way	A/W/S
Olympus	BF-XP160	2.8	60	1.2	90	2-way	Air
Olympus	BF-XP190	3.1	60	1.2	110	2-way (+rotary)	Air
Olympus	BF-MP190F	3.7	60	1.7	90	2-way (+rotary)	Air
Olympus	P190	4.2	60	2.0	110	2-way (+rotary)	Air
Olympus	H-Steri Scope Zero	2.3	60	No Channel	110	2-way	Air
Olympus	H-Steri Scope Slim	3.3	60	1.2	110	2-way	Air
Olympus	Spin Vision	4.0	73.5	2.0	110	2-way (rotary)	Air
Olympus	H-Steri Scope Normal	4.9	60	2.2	110	2-way	Air
Olympus	H-Steri Scope Large	5.8	60	2.8	110	2-way	Air
Pentax	EG16-K10	5.4	110	2.0	140	4-way	A/W/S
Pentax	EB-1575K	5.2	60	2.0	120	2-way	Air
Pentax	EB-1170K	3.7	60	1.2	120	2-way	Air
Pentax	EB-15-j10	5.4	60	2.0	120	2-way	Air
Pentax	EB-1170K	3.8	60	1.2	120	2-way	Air
Verathon	Ultraslim 2.8	2.8	56.6	No Channel	85 × 120	2-way	Air
Verathon	Slim 3.8	3.8	56.6	1.2	85 × 120	2-way	Air
Verathon	Regular 5.0	5.0	56.6	2.0	85 × 120	2-way	Air

A/W/S, air water suction buttons available.

The initial pediatric study used Olympus bronchoscopes (https://medical.olympusamerica.com/) for pediatric unsedated transnasal endoscopy, specifically the BF-XP160 (Outer Diameter 2.8 mm/Channel 1.2 mm) and the BF-MP160 (Outer Diameter 4.0 mm/Channel 2.0 mm) (Friedlander et al). Both of these allowed for sampling of the esophageal mucosa and had documented adequacy of sampling (1). These were used because the large diameter of the ultra-thin adult transnasal endoscopes made TNE intolerable for pediatric patients. Though both of these endoscopes were functional, they were older models and a subsequent study reported the use of the Olympus BF-XP190 (Outer Diameter 3.1 mm/Channel 1.2 mm) and Olympus BF-P190 (Outer Diameter 4.2 mm/Channel 2.0 mm). Full TN-EGD for monitoring of celiac disease in older pediatric patients has also been done using the Olympus N180 endoscope (Outer Diameter 4.9 mm/Channel 2.0) which is longer to allow visualization and biopsy of the duodenum (3). The limitations of the use of bronchoscopes as endoscopes include narrower field of view (90–110 degrees), shorter length of scope (60 cm length of bronchoscope vs. 110 cm in gastroscopes) which limits ability to fully evaluate the stomach and duodenum, 2 way tip deflection over 4 way deflection, a single valve to enable air/water, or suction. The bronchoscopes are also more likely to need frequent repair. Careful use and proper technique is important to minimize scope malfunction and optimize use. The thin outer diameter of the bronchoscope also allows for unsedated transgastrostomy endoscopy (TGE) via the gastrostomy and can be used to evaluate the esophagus (TG-Eso), stomach (TG-EG) and duodenum (TG-EGD). Other endoscope manufacturers such as Pentax (https://www.pentaxmedical.com/) and Fujinon (https://www.fujifilm.com/products/medical) also have ultra thin endoscopes and bronchoscopes that can be used with similar functions and limitations as described above.

Even prior to the COVID pandemic, efforts were made to optimize endoscopic efficiency and reduce risk of infection from reprocessed duodenoscopes (10). To remedy this, multiple manufacturers have been working on the development of single-use bronchoscopes, gastroscopes and duodenoscopes. Single Use Bronchoscopes have been developed for this purpose. Ambu (http://www.ambu.com) has developed several bronchoscopes (3.8 mm outer diameter/1.2 mm channel, 5.0 mm outer diameter/2.2 mm channel, 5.8 mm outer diameter/2.8 mm channel) that could be used for unsedated transnasal endoscopy in pediatrics, but have not been reported to date. Boston Scientific, Verathon, and Olympus have also released single-use bronchoscopes with various sizes and capabilities. Evoendo, Inc. (http://www.evoendo.com) recently launched a single-use gastroscope system (1.1 m length, 3.5 mm outer diameter/2 mm channel) with specific FDA clearance in pediatrics and is indicated for transnasal and transoral endoscopy of the upper gastrointestinal tract.

Each system has an associated control tower to integrate its system into a reporting system and image management. This is usually driven by each medical center's contract and electronic medical record.

As noted above when purchasing equipment, the repair rate and malfunction rate at the hospital should be noted to ensure adequate equipment availability and repair or replacement budget. Equipment sharing with pulmonology can be considered to minimize the cost of starting the program and to allow for sharing of equipment in case of equipment malfunction. The number of endoscopes available or purchased should also be considered depending on the rate of growth expected in a new program.

### Tissue sampling

One difference in pediatric transnasal endoscopy is the smaller diameter of the working channel which ranges 1.2–2.0 mm. This requires smaller biopsy forceps. Multiple studies in pediatric and adults have documented the adequacy of mucosal biopsies using ultra thin endoscopes or even thinner bronchoscopes (1, 8). For the 1.2 m channel, the endoscope manufacturer has special forceps designed including Boston Scientific (SpyGlass program), Olympus, or US Endoscopy. For a 2 mm channel, several forceps can be used including Boston Scientific, Olympus, MicroTec, or ConMed Forcep options include reusable forceps or single use forceps. Single use forceps can be more expensive, but convenient for disposal and do not require sterile processing. Reusable forceps can vary in price and require sterile processing with each use. If biopsies are not required, biopsy forceps are not needed and alters the level of processing required for the endoscope.

### Distraction equipment

Thoughtful consideration for methods to decrease anxiety, build rapport with the patient, and make the patient comfortable to cooperate with unsedated TNE is important. A distraction or dissociation method, such as Virtual Reality (VR) system, is important to the success of sedation-free endoscopy in pediatrics. Patients typically wear VR goggles during the entirety of the endoscopic procedure. This allows for patient distraction, analgesia and anxiolysis and the use of VR has been described in pediatric otolaryngology patients undergoing in-office nasal endoscopies (11). Multiple studies have shown its benefit in decreasing pain and distress for children and adults during medical procedures (12). Different VR systems and applications have been used and considerations for selecting a VR system include cost and fit, form and function for pediatric patients. Other techniques that have been used include watching television or listening to music. Some patients have opted out of wearing VR goggles during their procedure, which can also be successful if self-calming techniques are utilized. Additionally, CPT (Current Procedural Terminology) recently released coding for the physician work associated with virtual reality procedural dissociation (Table 3).

**Table 3:** Available CPT coding for unsedated transnasal endoscopy (13–15).

Description	CPT code
Transnasal Esophagoscopy	43197
Transnasal Esophagoscopy with biopsy	43198
Transnasal Esophagogastroscopy	0652T, 52 modifier
Transnasal Esophagogastroscopy with biopsy	0653, 52 modifier
Transnasal Esophagogastroduodenoscopy	0652T
Transnasal Esophagogastroduodenoscopy with biopsy	0653T
Transnasal Esophagogastroduodenoscopy with intraluminal tube/catheter placement	0654T
Transgastrostomy Esophagoscopy, Transgastrostomy Esophagogastroscopy or Transgastrostomy Esophagogastroduodenoscopy with biopsy	43499 (unlisted)
Virtual reality (VR) procedural dissociation services provided by the same physician or other qualified health care professional performing the diagnostic or therapeutic service that the VR procedural dissociation supports, requiring the presence of an independent, trained observer to assist in the monitoring of the patient's level of dissociation or consciousness and physiological status; initial 15 min of intraservice time, patient age 5 years or older	0771T
Virtual reality (VR) procedural dissociation services provided by the same physician or other qualified health care professional performing the diagnostic or therapeutic service that the VR procedural dissociation supports, requiring the presence of an independent, trained observer to assist in the monitoring of the patient's level of dissociation or consciousness and physiological status; each additional 15 min intraservice time	+0772T

### Patient preparation and topical analgesia

Although unsedated TNE does not require sedation or general anesthesia, patient preparation and topical analgesia is highly recommended to optimize the patient experience and comfort. TN-Eso is commonly reported to have a 2 h NPO time, and TN-EG or TN-EGD 4–6 h NPO time depending on the last ingested meal and rate of gastric emptying. Additionally, children who are undergoing TNE for the first time may benefit from recommendations to practice nasal sprays at home. This can include saline or their previously prescribed nasal corticosteroid. The authors have found this can help children be less anxious when administering 4% nasal lidocaine. The lidocaine is not palatable and can be stimulating to a child who never has had something sprayed in the nose before. Other topical lidocaine analgesia regimens are often customized by each center and sometimes include oxymetazoline. Table 4 lists lidocaine preparations commonly used.

**Table 4:** Lidocaine for topical analgesia^a,b,c,d^.

Type of administration	Dose administered	Total dose
Teleflex MADomizer (0.1 ml/spray)	4% Lidocaine: 3 sprays each nostril, 2 sprays oropharynx	0.8 ml (32 mg)
Nasal Atomizer (Variable Dosing with Syringe)	4% Lidocaine: 0.5 ml to each nostril 3 times	3–5 ml (120–200 mg)
Nasal Atomizer (Variable Dosing with Syringe) + Cotton Swab	4% Lidocaine: 0.5 ml to each nostril three times + cotton swab dipped in 4% lidocaine briefly touched to nasal meatus	3 ml (120 mg) + scant additional
Nasal Atomizer (Variable Dosing with Syringe) + Lidocaine Jelly	4% Lidocaine: 0.1–0.5 ml to each nostril three times + 2% Lidocaine jelly used as lubricant on scope shaft and/or cotton swab	Variable dosing
Syringe (Variable Dosing with Syringe)	2% lidocaine Jelly given via nostril and viscous lidocaine given orally based on weight	Variable dosing

^a^ Often Used Max Dose Lidocaine IV: 4 mg/kg/dose not exceed 300 mg.

^b^ 4% lidocaine (40 mg/ml).

^c^ Some centers also use Oxymetazoline as an adjunct to lidocaine.

^d^ Dosing based on practice from pediatric medical centers performing TNE.

### Other supplies

Other supplies for the procedure are necessary during TNE such as biopsy forceps, biopsy containers, endoscope valves, etc. These supplies are listed in Table 1B.

## Financial considerations

### Capital and consumable costs

Funding to start a TNE program can come from a variety of sources, but also has unique requirements depending on the goals and business plan of the program. Meeting with the business team can be helpful to map out the upfront costs (capital), recurring costs (consumables), staffing needs, programmatic projections, coding requirements, and marketing plans to grow the program. This greatly impacts the type or amount of funding required. For example, a program that purchases a large amount of capital equipment that sees few patients or does very few TNEs may not be financially successful. Also as noted above, if too few patients results in the inability to develop the optimal skill set required for the endoscopist, a subpar TNE experience for the patient could impact patient recruitment or programmatic development. Additionally, if coding and facility charges are not built or optimized for the program, this may halt programmatic growth and development. Capital (upfront) costs vary between the reusable endoscopic model and a single-use endoscopic model amongst individual hospitals and healthcare models. A list of capital equipment to estimate expenses are depicted in Table 1A. Purchasing new equipment and supplies may be very costly while availability of equipment already owned by the hospital or the possibility of equipment sharing amongst departments will decrease capital expenses, thus understanding the resources available in a center when creating a business plan is paramount. For example, use of equipment or procedure room sharing with pulmonology can decrease capital expenses. The decision between a reusable system or a single use system is center-dependent, as each system will require varying capital costs, repair or replacement endoscope budgeting, sterile process budgeting, and varying consumable equipment costs. An important consideration is to ensure that the program selected will allow for a successful, efficient TNE program where funding that is generated from the program can then help with additional programmatic growth. Planning with the business team should entail a detailed comparison of center-specific capital costs, anticipated repair/replacement budgeting and consumable costs of each type of system prior to equipment selection and purchasing to ensure financial success of a new TNE program. To start a TNE program, 4 reusable scopes or a month's supply of single-use endoscopes is recommended to allow for optimal efficiency and clinic flow. Purchasing a single endoscope potentially could limit efficiency, therefore a minimum of 2 endoscopes, to allow for back-up in case of technical difficulties with the available equipment, is recommended.

### Business plan

A business plan should serve as a roadmap on how to structure, maintain and grow the TNE program. Therefore, it should incorporate important considerations including number of endoscopies anticipated per year, how to integrate into practice (as part of clinic sessions or separate procedure sessions), staffing needs, growth potential year over year, provider referral base, ongoing costs, potential patients, value to the practice, recognition for the hospital or healthcare system, financial benefit, research potential, methods to grow the program, and anticipated time to reach the break-even revenue point or positive revenue points for the program.

### Capacity & efficiency

**Figure 2:**
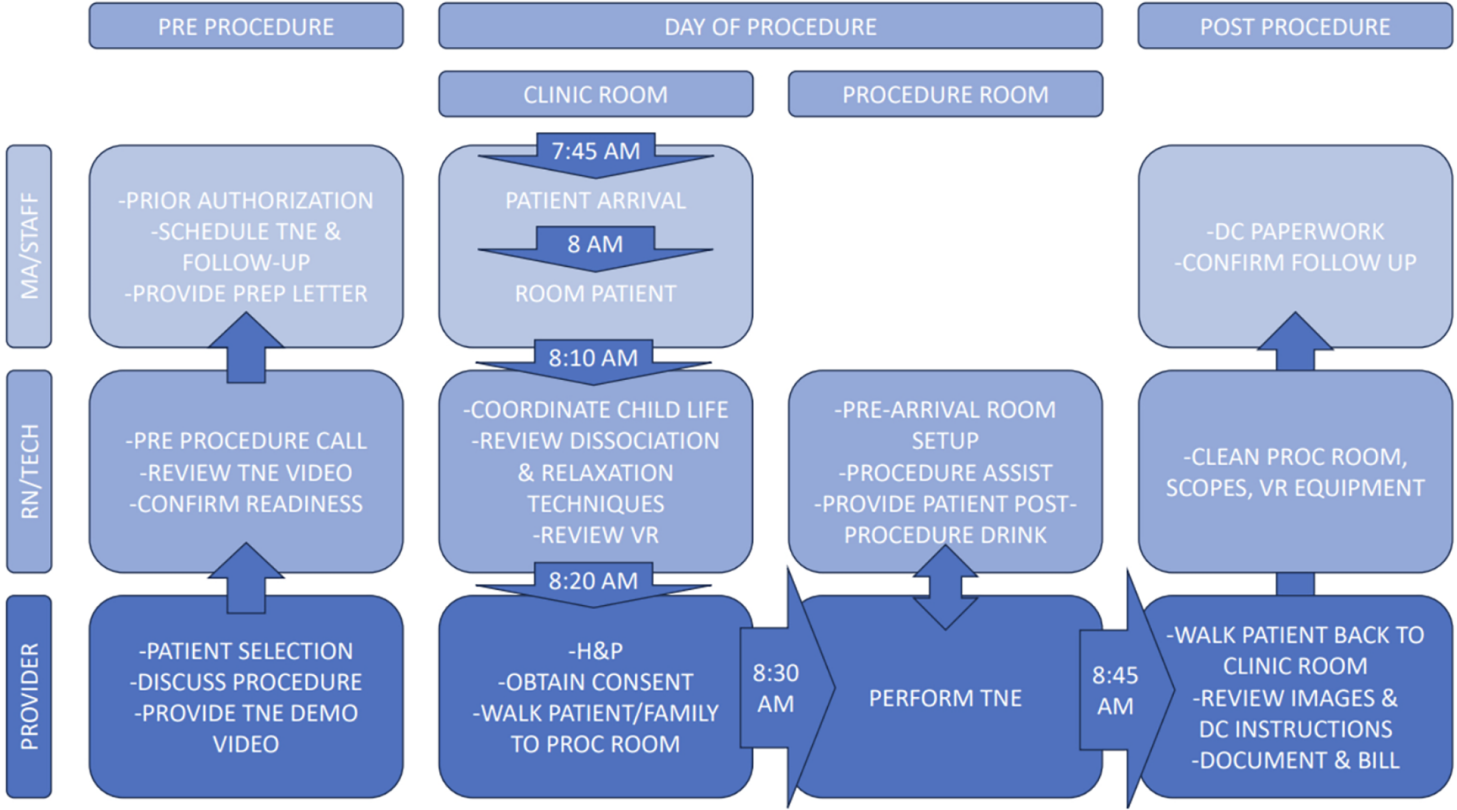
Workflow for TNE.

The capacity of an sedation-free endoscopy program is generally limited by space, room turnover, and available equipment/endoscopes. When initially starting TNE, 60–90 min visits should be adequate time from check in to discharge. As programs become more experienced, 30–40 min visits are reasonable, allowing for up to 6–8 unsedated endoscopies per 4 h clinic session. A patient's first time undergoing TNE typically requires more time as they often benefit from preparation with a child life specialist and require more anticipatory guidance. The suggested workflow is listed in Figure 2. The approximate time for procedure preparation is 20–30 min and time in the procedure room is 10–15 min.

## Billing & coding

### Chargemaster and CPT coding

The reimbursement for sedation-free endoscopy in a hospital outpatient department in the United States revolves around two components: 1) the facility charge and 2) the physician charge (CPT code). The facility charge includes cost of the supplies (see Table 1B), the endoscopes (which would include either the cost of the reusable endoscope spread out over time and repair/re-processing costs or the cost of the-single use endoscope), staffing, and room which should be carefully calculated and developed in order to ensure a financially successful program.

The CPT code is the physician charge for the procedure. Current available CPT codes for transnasal endoscopy of the upper gastrointestinal tract are in Table 3. As noted above, additional codes are now available when VR distraction and dissociation is used (see Table 3). There are specific requirements for use of these codes. These codes are specific to billing in the United States.

### Ambulatory surgery center (ASC) vs. outpatient hospital vs. clinic space

Coding and reimbursement in the United States is highly dependent on the licensing of each area and subject to requirements. The decision to have the sedation-free endoscopy unit in an ASC/Procedure Unit, Outpatient Hospital Clinic, or a general outpatient clinic will have ramifications on reimbursement. Generally, a procedure unit at a hospital facility and or the hospital facility outpatient environment allows for a facility charge, but lower specific physician reimbursement. Performing unsedated endoscopy in an independent ASC may have lower facility reimbursement. If it is done in a general outpatient non-hospital (non-hospital facility) clinic, the procedure may allow for higher specific physician reimbursement, but inability to charge a facility charge. The decision by each medical center of where to locate the procedure is dependent on each location's clinical needs, strategic plans, and space availability.

## Special considerations

### Parent/caregiver preparation

Most institutions that perform pediatric TNE allow parents/caregivers to be in the room, as they can provide reassurance and comfort to the child during the unsedated procedure. Many parents/caregivers are interested in being able to observe their child's endoscopy. Anticipatory guidance about the procedure is of utmost importance as some families may be nervous around blood or biological tissues. Consideration of a pre-op visit, phone call, or pre-procedure review of video and handouts can be helpful (2). The pre-op visit or call is often where nil per os (NPO) recommendations and any preparation requirements are given. Additionally this call or visit can be done either by the physician or medical staff, can address questions and concerns the parents/caregivers may have and allows for anticipatory guidance.

### Procedural documentation

**Figure 3:**
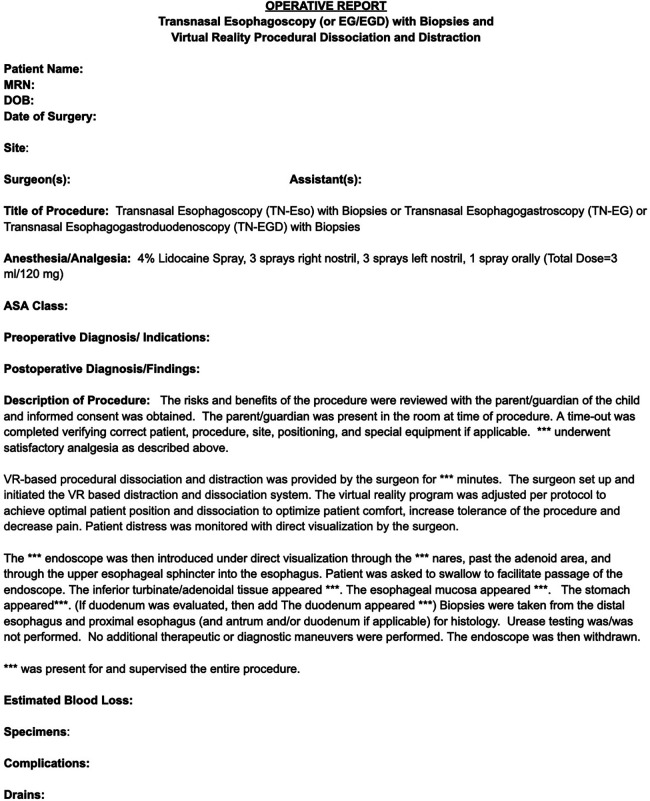
Sample note template.

Documentation for unsedated endoscopy is similar to sedated endoscopy but requires special addendums for use of the specific billing codes (Figure 3). Specific differences include visualization of the turbinates, adenoids, and pharynx to differentiate oral from nasal endoscopy. Documentation for nursing staff may vary by institution but can include medications, patient vital signs, equipment used, and billing of supplies.

## Training considerations

### Learning TNE

Learning unsedated transnasal endoscopy is similar to learning endoscopy. It requires motivation, patience, procedural frequency, and a willing teacher. On top of learning the basic procedural technique, learning patient/family management and how different patients respond and behave during a sedation-free procedure is also needed. Although most patients do well, some require more reassurance. When first starting TNE, learning how to perform a procedure while talking and reassuring the patient may take some practice. Based on the authors' experience, completing the first 10–20 cases in a short period of time (1–3 months) is beneficial to achieve proficiency. Additionally, collaboration amongst centers and discussing with colleagues currently performing TNE is helpful when first starting as it can manage expectations and can alleviate physician concerns and nervousness around performing unsedated TNE. A suggested learning process for TNE:

1. Learn nasal anatomy.
2. Consider practicing placement of feeding style nasogastric tube or impedance probe at least 10 times on unsedated patient with nursing staff to familiarize with unsedated procedures and UES intubation in an awake patient
3. Learn laryngoscopy or TNE via training model or via mentorship. Consider partnering with pulmonology or ENT.
4. Attempt TNE focusing on technique and laryngoscopy, esophageal intubation and biopsy
5. Perform TNE on live patients at least 1–2 times weekly, but no less than 2 times per month to achieve mastery. Mastery usually with >100 TNE. If less than 2 times per month mastery may take considerably longer.

When performing transnasal endoscopy an endoscopist will need to learn nasal anatomy and variant endoscopic techniques that are associated with an ultra-slim endoscope. Therefore, working with an otolaryngologist can be beneficial (16). Three-dimensional models can be used as simulators to help new learners, particularly with nasal intubation. Patient simulators may be available commercially from various manufacturers or may already be available within your facility as part of code training equipment on site. The art of unsedated esophageal intubation is to provide gentle pressure while asking the patient to swallow after passing through the upper esophageal sphincter, similar to how one would place an NG feeding tube. However, the endoscope is stiffer than an NG feeding tube, therefore requires more precise placement and direct visualization to avoid tissue abrasion and endotracheal intubation. Most endoscopists will develop minimal proficiency around 5–10 procedures, and most master the procedure once they have performed 100 TNEs. Endoscopists who are competent in conventional EGD can become competent in unsedated TNE, even without structured training (16). Obtaining biopsies of the esophagus using smaller forceps can require practice and could be practiced during conventional EGD by not utilizing air insufflation and when the esophagus is collapsed. Performing TN-EG or TN-EGD also requires practice and a different technique due to the patient being in a sitting position and the dimmer light of a thinner scope.

Learning unsedated TG-Eso, TG-EG, or TG-EGD requires placing the endoscope via the gastrostomy tube, advancing the endoscope, and obtaining biopsies at the desired location. Visualization of the stomach and duodenum is similar to conventional EGD. Esophageal intubation retrograde can be challenging. The lower esophageal sphincter can be visualized by locating a dimple of tissue with intermittent saliva being extruded. An older child can also swallow clear but colored liquid to help facilitate locating the lower esophageal sphincter. A 12 french or 14 french gastrostomy tube will allow passage of an endoscope with an outer diameter of 4.2 mm endoscope. A 16 french gastrostomy tube will allow passage of an endoscope with an outer diameter of 5 mm.

## Review

In conclusion, the use of sedation-free endoscopy is expanding around the United States and there is growing interest from physicians, professional societies, patient advocacy groups and patients for use in pediatrics. It has transformative potential in pediatric gastroenterology as it provides for a more efficient, safe, and cost-effective method to evaluate and follow up pediatric disorders of the upper gastrointestinal tract. Development of an unsedated pediatric endoscopy program will improve patient care, decrease the need for anesthesia, provide a lower cost and safe alternative to traditional sedated endoscopy, and is a viable component to a pediatric gastroenterology practice.
